# Impact of community tracer teams on treatment outcomes among tuberculosis patients in South Africa

**DOI:** 10.1186/1471-2458-12-621

**Published:** 2012-08-07

**Authors:** Liza E Bronner, Laura J Podewils, Annatjie Peters, Pushpakanthi Somnath, Lorna Nshuti, Martie van der Walt, Lerole David Mametja

**Affiliations:** 1 Division of TB Elimination, Centers for Disease Control and Prevention, 1600 Clifton Road NE Mailstop E-10, Atlanta, GA, 3033, USA; 2 Global AIDS Program, Centers for Disease Control and Prevention, 877 Pretorius Street, Arcadia, 0007, South Africa; 3 TB Epidemiology and Intervention Research Unit, South African Medical Research Council, 1 Soutpansberg Road, Private Bag X385, Pretoria, 0001, South Africa; 4 Tuberculosis Control and Management, Republic of South Africa National Department of Health, Private Bag X828, Pretoria, 0001, South Africa

**Keywords:** Default, Community mobilization, Treatment adherence, Outreach

## Abstract

**Background:**

Tuberculosis (TB) indicators in South Africa currently remain well below global targets. In 2008, the National Tuberculosis Program (NTP) implemented a community mobilization program in all nine provinces to trace TB patients that had missed a treatment or clinic visit. Implementation sites were selected by TB program managers and teams liaised with health facilities to identify patients for tracing activities. The objective of this analysis was to assess the impact of the TB Tracer Project on treatment outcomes among TB patients.

**Methods:**

The study population included all smear positive TB patients registered in the Electronic TB Registry from Quarter 1 2007-Quarter 1 2009 in South Africa. Subdistricts were used as the unit of analysis, with each designated as either tracer (standard TB program plus tracer project) or non-tracer (standard TB program only). Mixed linear regression models were utilized to calculate the percent quarterly change in treatment outcomes and to compare changes in treatment outcomes from Quarter 1 2007 to Quarter 1 2009 between tracer and non-tracer subdistricts.

**Results:**

For all provinces combined, the percent quarterly change decreased significantly for default treatment outcomes among tracer subdistricts (−0.031%; p < 0.001) and increased significantly for successful treatment outcomes among tracer subdistricts (0.003%; p = 0.03). A significant decrease in the proportion of patient default was observed for all provinces combined over the time period comparing tracer and non-tracer subdistricts (p = 0.02). Examination in stratified models revealed the results were not consistent across all provinces; significant differences were observed between tracer and non-tracer subdistricts over time in five of nine provinces for treatment default.

**Conclusions:**

Community mobilization of teams to trace TB patients that missed a clinic appointment or treatment dose may be an effective strategy to mitigate default rates and improve treatment outcomes. Additional information is necessary to identify best practices and elucidate discrepancies across provinces; these findings will help guide the NTP in optimizing the adoption of tracing activities for TB control.

## Background

Tuberculosis (TB) is a leading cause of morbidity and mortality worldwide, infecting an estimated 9.4 million persons and causing death in 1.7 million persons annually [1]. The World Health Organization (WHO) ranks South Africa as having the third highest TB incidence rate among the top 22 high-burden TB countries, with an estimated 405,982 persons diagnosed with TB each year (incidence rate 971/100,000) [1].

Patient default from treatment is one of the most important problems in TB control [2,3]. In 1996, the South Africa National Tuberculosis Program (NTP) adopted the Directly Observed Treatment Short-Course (DOTS) strategy nationwide for the treatment of TB patients. While the NTP has implemented several strategies over the past decade to improve access to treatment and support treatment compliance among TB patients, at 76% the treatment success rate remains well below WHO targets of 85% cured or completing treatment necessary to mitigate the spread of TB [1,4-6].

Default from TB treatment poses a serious health risk to TB-infected individuals and to the community. The number of TB patients who default from TB treatment in South Africa, defined as missing at least 2 consecutive months of treatment [6], remains high ranging from 5.9 – 14.7% [1,4]. TB treatment defaulters, especially those who are smear positive, propagate ongoing community transmission and promote the development and acquisition of drug-resistant TB strains resulting in a higher number of TB cases [3,7,8]. Previous studies have shown that over one-third of patients who default from treatment are culture-positive for TB and therefore infectious at the time of default [3,7]. Additionally, research in India found that patients who defaulted from treatment had a standardized mortality ratio of 14.3 versus 2.0 in patients who completed treatment [9].

Research has shown that TB patient tracing activities are an effective method to significantly reduce TB treatment default [8,10,11]. However, there is little research documenting the effect of tracing on TB treatment outcomes [11]. In 2008, the South Africa NTP initiated a national project (hereafter referred to as the TB Tracer Project) aiming to decrease default rates and improve patient outcomes through community mobilization. The aim of this study is to evaluate the impact of the TB Tracer Project on TB treatment outcomes in South Africa.

## Methods

### TB Tracer Project design

**Figure 1:**
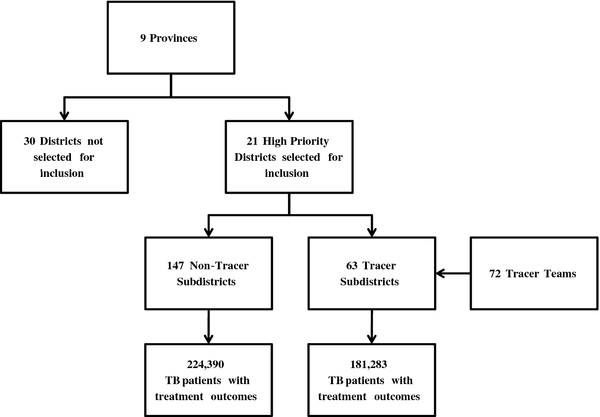
**Overview of the TB Tracer Project implementation and study population of TB patients registered in the ETR included for analysis (n = 405,673).** The South African National TB Program selected 2 to 4 districts from each of the 9 provinces of South Africa for inclusion in the TB Tracer Project. The selected districts were those with the highest rates of treatment default in 2006. The districts then selected four to six subdistricts to carry out the project with at least one tracer team assigned to each selected subdistrict.

The TB Tracer Project was implemented from January 2008 to May 2009 in all nine provinces of South Africa. Two to four districts in each province deemed as high priority by the South African NTP with the highest rates of TB treatment default in 2006 were selected for inclusion [12]. Each district then selected four to six subdistricts to carry out the project. Each subdistrict was assigned at least one dedicated TB tracer team comprised of one registered nurse, two community health care workers, and one data capturer. Teams of health care workers were employed at health facilities (i.e. hospitals, clinics, and community health centers) to trace TB patients who had interrupted treatment or had missed a clinic appointment to obtain a sputum sample to evaluate their smear status for TB. Since the project was implemented as a programmatic intervention, tracer team activities, mechanisms of tracing, modes of transportation, and health facility placement varied by subdistrict. Over the course of the project there were 21 districts selected for inclusion with 63 tracer subdistricts with 72 project-designated tracer teams that participated during the project period and 147 non-tracer subdistricts; there were 30 districts that were not selected for inclusion in the project (Figure 1). 

### Study design

This retrospective study was conducted using routinely collected data from the South African national database for TB surveillance, the Electronic TB Registry (ETR). Aggregate TB patient data is recorded quarterly in the ETR at the subdistrict level; therefore, the subdistrict level was used as the unit of analysis and time was measured quarterly in this study. The study population included all smear positive TB patients registered with final treatment outcomes recorded in the National ETR from Quarter 1 2007 through Quarter 1 2009 (Q1 to Q4 2007, Q1 to Q4 2008, and Q1 2009) across the nine provinces of South Africa.

### Definitions and outcomes

ETR data from Q1 through Q4 2007 was included in the analysis to provide information on treatment outcomes prior to the implementation of the TB Tracer Project (Q1 2008 through Q1 2009) and to allow for the analysis of the change in trend of TB treatment outcomes over time. Tracer subdistricts were considered as those where at least one health facility included TB team tracing activities in addition to standard NTP patient services, whereas non-tracer subdistricts provided only standard NTP patient services.

The proportion of patients with treatment outcomes registered in the National ETR as cured, completed, defaulted, failed, and died were each evaluated separately as the primary impact indicators for comparing patients from tracer and non-tracer subdistricts. Completion and cure treatment outcomes were combined to define a successful treatment outcome as an additional primary impact indicator for comparison with all other treatment outcomes (default, failed, and died). Patients registered in the National ETR with missing treatment outcome data were excluded from this analysis.

### Statistical analysis

Descriptive statistics were used to summarize characteristics of the population of TB patients registered in the ETR for the time period evaluated (Q1 2007 through Q1 2009). Longitudinal analysis was utilized to evaluate changes in each TB treatment outcome between tracer and non-tracer subdistricts over time (using PROC GLIMMIX in SAS). Each outcome of interest (default, success, cured, completed, failed, and died) was evaluated separately, using the log of the proportion of each outcome at each time point. Proportions were calculated by using the counts of patients recorded in the ETR with a given outcome as the numerator divided by the total number of patients in the ETR with a final treatment outcome recorded. The percent quarterly change over time for each TB treatment outcome was computed for each the tracer and non-tracer subdistricts for all smear positive cases and for all smear positive cases in each province.

Mixed linear regression models were used with a random intercept that specified the province variable as a random cluster effect to account for spatial correlation of TB treatment outcomes within provinces of South Africa. The tracer indicator (tracer vs. non-tracer subdistricts) and time variable (continuous variable measured quarterly) were held as fixed effects in the model and the tracer*time interaction term was included to assess the effect of the tracer teams over time. Province stratified analyses were conducted for the two primary outcomes of interest, default and treatment success, using the same model parameters with the exception of province clusters. The p-value for significance of the tracer status by time interaction term is reported to assess change in the linear trend of each treatment outcome comparing tracer versus non-tracer subdistricts over time. Statistical significance was considered at a p-value<0.05. All analyses were conducted using SAS version 9.3 (SAS Institute Inc., Cary, NC, USA).

### Ethical considerations

This evaluation was approved by the Institutional Review Boards of the U.S. and South African Centers for Disease Control and Prevention and the South African Medical Research Council. Information was derived from existing electronic data systems that are part of routine monitoring and evaluation of the NTP. No patients were contacted as part of this analysis, and the data abstraction did not involve individual patient charts or information.

## Results

### Study population characteristics

From Q1 2007 to Q1 2009, there were 405,673 smear positive TB patients registered in the National ETR with treatment outcomes recorded (18,275 TB patients missing treatment outcome data were excluded from this analysis). Of these patients, 45% (181,283) received TB health services in subdistricts where TB tracer teams were operating (Table 1). New patients accounted for 75% (303,846) of TB patients in the ETR database during the project period. The greatest proportion of TB patients were from Kwazulu-Natal (85,634; 21%), Eastern Cape (72,371; 18%), and Western Cape (73,874; 18%) Provinces. Among the 405,673 TB patients analyzed, 64% (260,219)had a final treatment outcome of cured, yet 10% (38,783) defaulted from TB treatment. When comparing patients from tracer subdistricts to those from non-tracer subdistricts, 60% (108,439) versus 68% (151,780) patients were cured; whereas, 11% (20,538) versus 8% (18,245) patients defaulted, respectively.

**Table 1:** Characteristics of TB patients in the Electronic TB Registry for Tracer and Non-Tracer subdistricts, Quarter 1 2007-Quarter 1 2009, South Africa

**Characteristic**	**All Patients**		**Tracer**		**Non-Tracer**	
	**(n = 405, 673)**		**(n = 181,283)**		**(n = 224,390)**	
	**n**	**%**	**n**	**%**	**n**	**%**
Case type						
New	303846	74.9	141166	77.9	162680	72.5
Retreatment	101827	25.1	40117	22.1	61710	27.5
Province Totals^						
Eastern Cape	72371	17.8	37840	20.9	34531	15.4
Free State	26920	6.6	9457	5.2	17463	7.8
Gauteng	58036	14.3	32769	18.1	25267	11.3
Kwazulu-Natal	85634	21.1	46517	25.7	39117	17.4
Limpopo	19281	4.8	7126	3.9	12155	5.4
Mpumalanga	26623	6.6	16083	8.9	10540	4.7
Northern Cape	12541	3.1	5636	3.1	6905	3.1
Northwest	30393	7.5	15436	8.5	14957	6.7
Western Cape	73874	18.2	10419	5.7	63455	28.3
Treatment Outcomes*,‡^						
Defaulted	38783	9.6	20538	11.3	18245	8.1
Cured	260219	64.1	108439	59.8	151780	67.6
Completed	40276	9.9	20579	11.4	19697	8.8
Failed	8885	2.2	4199	2.3	4686	2.1
Died	34355	8.5	16330	9.0	18025	8.0
MDR-TB	2033	0.5	581	0.3	1452	0.6
Transferred	21122	5.2	10617	5.9	10505	4.7

†The Electronic TB Registry (ETR) is the national TB surveillance database used in South Africa.

*Treatment success was defined as having a registered treatment outcome of either ‘Cured’ or ‘Completed’ in the ETR (n = 300,495; Tracer n = 129,018; Non-Tracer n = 171,477).

‡Patients registered in the National ETR database with missing treatment outcome data (n = 18,275; Tracer n = 10,292; Non-Tracer n = 7,983) were considered as missing and were excluded from this analysis.

^Percentages total to greater than 100% due to rounding of percentage values.

### Percent quarterly change for all treatment outcomes: all smear positive TB patients

For all smear positive TB patients, a significant decrease in the percent quarterly change in default treatment outcomes of −0.031% was observed in tracer subdistricts (p < 0.001) compared to a decrease of only −0.002% in non-tracer subdistricts (p = 0.85) (Table 2). Additionally, a significant increase in the percent quarterly change in successfully treatment outcomes was observed in tracer subdistricts (tracer = 0.003%, p = 0.03; non-tracer = 0.002%, p = 0.16). The percent quarterly change in cured treatment outcomes increased significantly in the tracer and non-tracer subdistricts (tracer = 0.007%, p < 0.01; non-tracer = 0.010%, p < 0.001); by contrast, there was a significant decrease in completion treatment outcomes in both groups (tracer = −0.029%, p < 0.01; non-tracer = −0.059%, p < 0.001).

**Table 2:** Percent quarterly change in proportion of TB treatment outcomes, Tracer vs. Non-Tracer subdistricts, Q1 2007-Q1 2009, South Africa

	**Tracer**			**Non-Tracer**		
	**Percent quarterly change, %**^†^	**95% CI**	**P-value**	**Percent quarterly change, %**^†^	**95% CI**	**P-value**
Default	−0.031	(−0.048, -0.014)				<0.001	−0.002	(−0.019, 0.016)	0.85
Success*	0.003	(0.001, 0.006)				0.03	0.002	(−0.001, 0.004)	0.16
Cure	0.007	(0.002, 0.012)				<0.01	0.010	(0.005, 0.014)	<0.001
Completed	−0.029	(−0.047, -0.011)				<0.01	−0.059	(−0.076, -0.041)	<0.001
Failed	0.012	(−0.014, 0.037)				0.36	−0.030	(−0.054, -0.006)	0.01
Died	0.002	(−0.012, 0.016)				0.79	−0.006	(−0.019, 0.007)	0.34

†Calculations of percent quarterly change included all smear positive TB patients registered with treatment outcome data in the ETR.

*Treatment success was defined as having a registered treatment outcome of either ‘Cured’ or ‘Completed’ in the ETR and was calculated by combining ‘Cured’ and ‘Completed’ treatment outcomes.

### Analysis of trends in treatment outcomes: all smear positive TB patients

**Figure 2:**
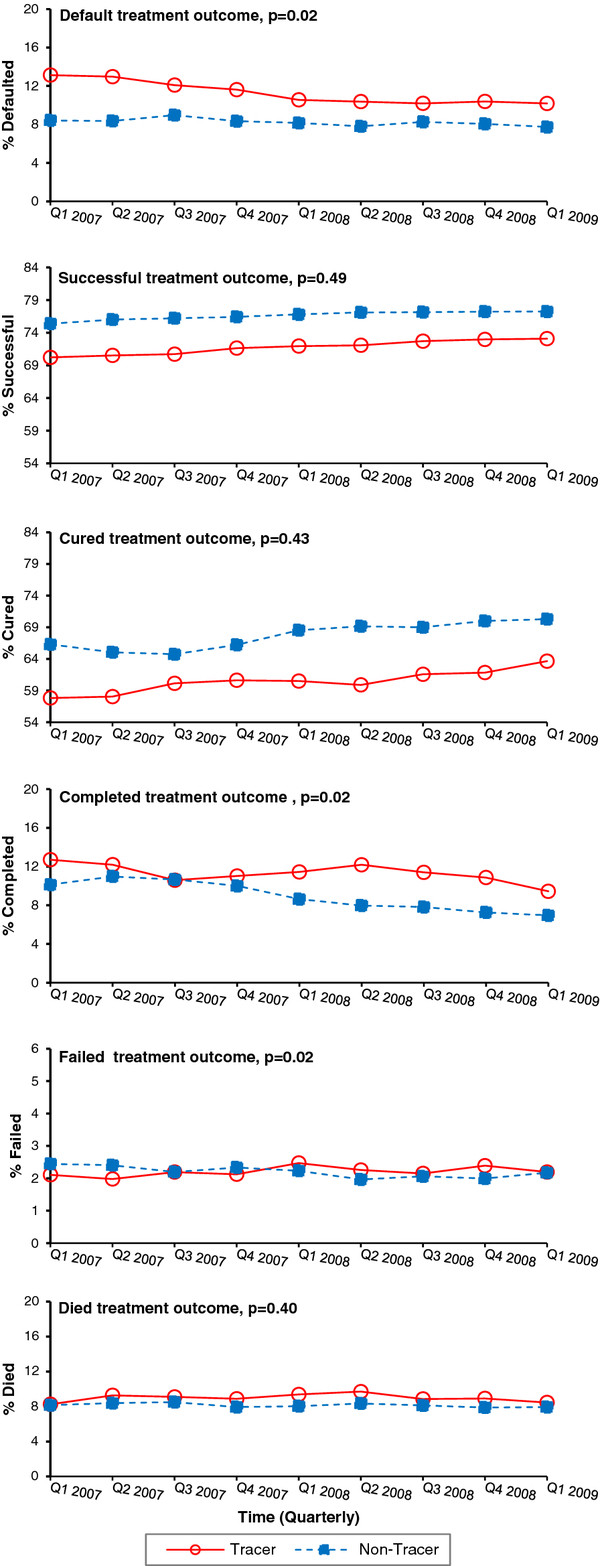
**Proportion of all smear positive TB patients with final treatment outcomes for all provinces, tracer vs. non-tracer subdistricts, Quarter 1 2007-Quarter 1 2009, South Africa.** A significant difference was detected in tracer subdistricts (solid line) compared to non-tracer subdistricts (dashed line) in the proportion of treatment outcomes for patient default, completion, and failure among all smear positive TB patients with treatment outcomes recorded in the ETR. The p-value reported for each graph represents the significance of the tracer status by time interaction term to assess the change in linear trend of each treatment outcome comparing tracer versus non-tracer subdistricts over time**.** The y-axis of each graph in Figure 2 varies according to the baseline treatment outcome recorded for Q1 2007. The y-axes were not standardized on a 0-100% scale to allow for better visualization of the percent change in each treatment outcome from baseline to the end of the evaluation period in Q1 2009.

When comparing the change in proportions of treatment outcomes in tracer versus non-tracer subdistricts from Q1 2007 to Q1 2009, significant changes over time were detected in the proportions of defaulted, completed, and failed treatment outcomes (Figure 2). The proportion of patients who defaulted from treatment in subdistricts with tracer teams declined from 13.1% in Q1 2007 to 10.2% in Q1 2009, a decrease that was significantly greater than observed in non-tracer subdistricts from 8.4% to 7.7% (p-value for tracer indicator by time interaction*,* p = 0.02). The proportion of TB patients with a successful treatment outcome increased in the tracer subdistricts (70.5% to 73.1%) compared to the non-tracer subdistricts (76.4% to 77.2%), but this change was not significant over time (interaction p = 0.49). Meanwhile, the proportion of treatment completion decreased significantly from 12.7% to 9.4% in tracer subdistricts versus 10.1% to 6.9% in non-tracer subdistricts (interaction p = 0.02). Further, a small but significant increase in the proportion of treatment failure was observed in the tracer subdistricts (2.1% to 2.2%) versus non-tracer subdistricts (2.4% to 2.2%) (interaction p = 0.02).

### Analysis of treatment default: all smear positive TB patients stratified by province

**Figure 3:**
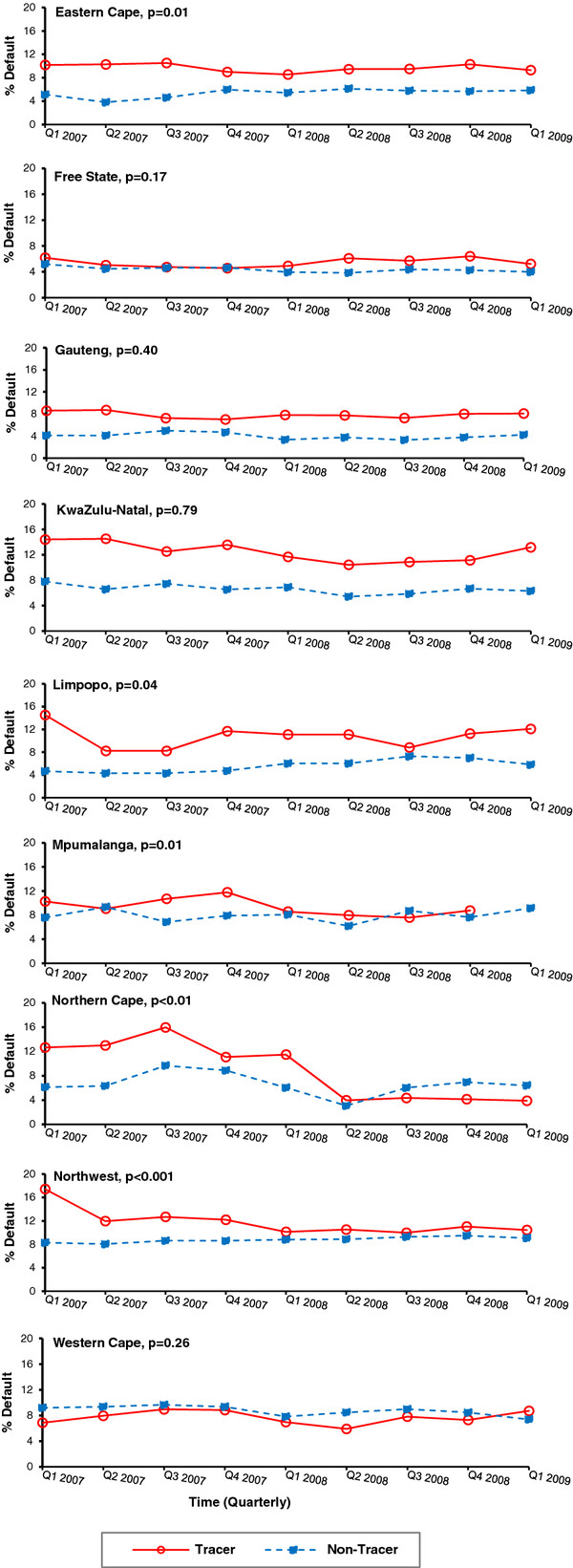
**Proportion of all smear positive TB patients with default TB treatment outcomes stratified by province, tracer vs. non-tracer subdistricts, Q1 2007-Q1 2009, South Africa.** A significant difference was detected in the proportion of treatment default in 5/9 provinces in South Africa in tracer subdistricts (solid line) compared to non-tracer subdistricts (dashed line). The p-value reported for each graph represents the significance of the tracer status by time interaction term to assess the change in linear trend of each treatment outcome comparing tracer versus non-tracer subdistricts over time. The y-axis representations for percentages of treatment default were not standardized on a 0-100% scale to allow for better visualization of the percent change from Q1 2007 to the end of the evaluation period in Q1 2009.

Province stratified models for default treatment outcomes among all TB cases demonstrated inconsistent results across the nine provinces. The tracer subdistricts in four of nine provinces displayed a significant decrease in the percent quarterly change in patient default; the non-tracer subdistricts in three different provinces and in one of the same provinces (KwaZulu-Natal) also revealed a significant decline (Table 3). However, the interaction of the tracer teams over time demonstrated a significant decrease in the proportion of patient defaultin five provinces for tracer versus non-tracer subdistricts (Figure 3). The proportion of patient default among tracer subdistricts decreased significantly in Eastern Cape (10% to 9%), Limpopo (14.5% to 12.1%), Mpumalanga (10% to 5%), Northern Cape (13% to 4%), and Northwest (17% to 10%) Provinces. Conversely, the non-tracer subdistricts from the same provinces showed an increase in the proportion of default treatment outcomes during the analysis time period.

**Table 3:** Percent quarterly change in proportion of default TB treatment outcomes stratified by province, Tracer vs. Non-Tracer subdistricts, Q1 2007-Q1 2009, South Africa

**Province**	**Tracer**			**Non-Tracer**		
	**Quarterly change in default,%**^†^	**95% CI**	**P-value**	**Quarterly change in default,%**^†^	**95% CI**	**P-value**
Eastern Cape	−0.009	(−0.025, 0.008)	0.29	0.030	(0.007, 0.053)	0.01
Free State	0.008	(−0.031, 0.048)	0.65	−0.026	(−0.060, 0.008)	0.12
Gauteng	−0.005	(−0.026, 0.015)	0.57	−0.020	(−0.049, 0.009)	0.17
Kwazulu-Natal	−0.032	(−0.053, -0.009)	<0.01	−0.027	(−0.053, -0.002)	0.04
Limpopo	−0.003	(−0.043, 0.036)	0.86	0.059	(0.015, 0.102)	0.01
Mpumalanga	−0.060	(−0.096, -0.024)	<0.01	0.009	(−0.027, 0.046)	0.60
Northern Cape	−0.191	(−0.270, -0.112)	<0.001	−0.025	(−0.099, 0.049)	0.48
Northwest	−0.055	(−0.079, -0.031)	<0.001	0.017	(−0.011, 0.045)	0.22
Western Cape	0.002	(−0.041, 0.046)	0.91	−0.023	(−0.039, -0.007)	<0.01

†Calculations of percent quarterly change included all smear positive TB patients registered with treatment outcome data in the ETR.

### Analysis of treatment success: all smear positive TB patients stratified by province

**Figure 4:**
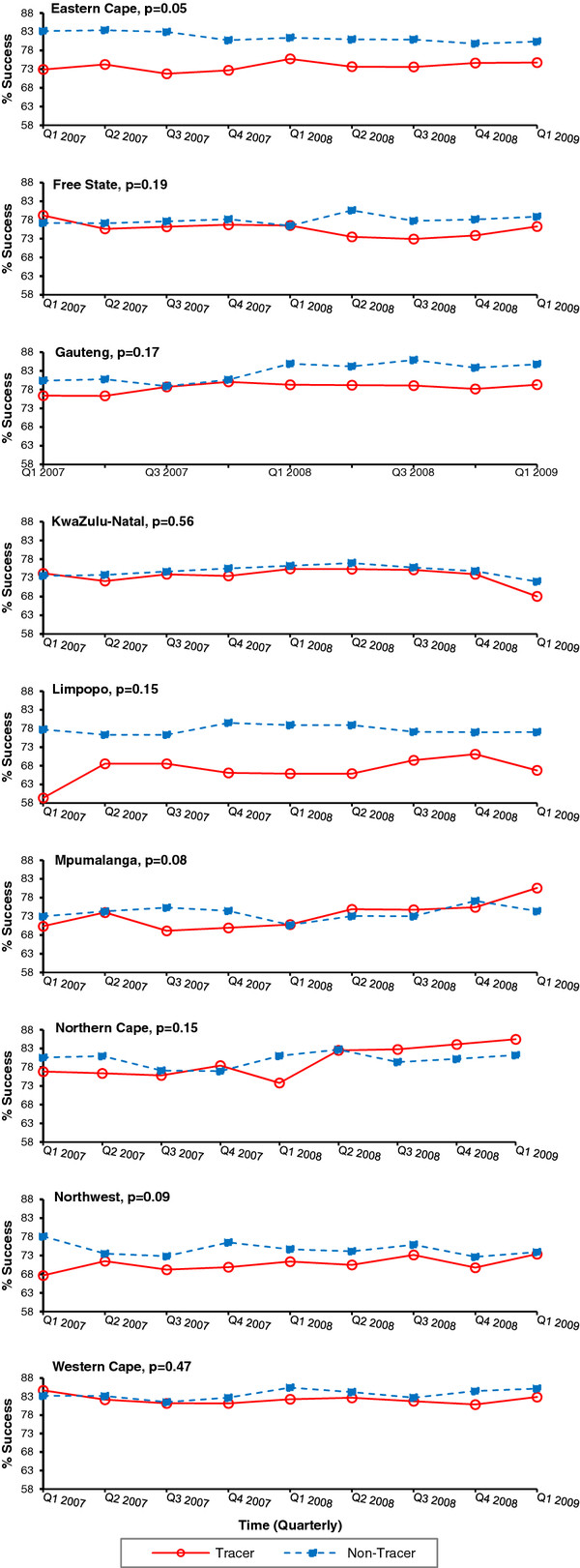
**Proportion of all smear positive TB patients with successful TB treatment outcomes stratified by province, tracer vs. non-tracer subdistricts, Q1 2007-Q1 2009, South Africa.** A significant difference was detected in the proportion of treatment success in 1/9 provinces in South Africa in tracer subdistricts (solid line) compared to non-tracer subdistricts (dashed line). The p-value reported for each graph represents the significance of the tracer status by time interaction term to assess the change in linear trend of each treatment outcome comparing tracer versus non-tracer subdistricts over time. The y-axis representations for percentages of treatment success were not standardized on a 0-100% scale to allow for better visualization of the percent change from Q1 2007 to the end of the evaluation period in Q1 2009.

The stratified analysis exposed similar discrepancies in the results of the tracer teams on successful treatment outcomes. A significant increase in the percent quarterly change of successful treatment outcomes occurred in two of nine provinces for tracer subdistricts and in one province for non-tracer subdistricts (Table 4). When examining the change in treatment success over time in tracer versus non-tracer subdistricts, only Eastern Cape Province displayed results that approached significance (interaction p = 0.05) (Figure 4). Nonetheless, the proportion of treatment successincreased from Q1 2007 to Q1 2009 among tracer subdistricts in Eastern Cape (73% to 75%), Gauteng (76% to 79%), Limpopo (59% to 67%), Mpumalanga (70% to 81%), Northern Cape (77% to 86%), and Northwest (68% to 73%) Provinces. Additionally, among the non-tracer subdistrictsin Eastern Cape,the success rate declined from 83% to 80% and in Northwest Province from 78% to 74%. Meanwhile, Free State Province demonstrated a decrease in treatment success among the tracer subdistricts while treatment success increased in non-tracer subdistricts (interaction p = 0.19). Kwazulu-Natal Province displayed a similar decrease in treatment success in both the tracer and non-tracer subdistricts.

**Table 4:** Percent quarterly change in proportion of successful TB treatment outcomes stratified by province, Tracer vs. Non-Tracer subdistricts, Q1 2007-Q1 2009, South Africa

**Province**	**Tracer**			**Non-Tracer**		
	**Quarterly change in success, %**^†^	**95% CI**	**P-value**	**Quarterly change in success, %**^†^	**95% CI**	**P-value**
Eastern Cape	0.003	(−0.003, 0.009)	0.29	−0.005	(−0.011, 0.001)	0.08
Free State	−0.006	(−0.016, 0.005)	0.25	0.003	(−0.006, 0.011)	0.51
Gauteng	0.003	(−0.002, 0.009)	0.23	0.009	(0.003, 0.015)	<0.01
Kwazulu-Natal	−0.001	(−0.006, 0.005)	0.75	0.001	(−0.004, 0.007)	0.61
Limpopo	0.011	(−0.002, 0.024)	0.10	−0.001	(−0.010, 0.009)	0.86
Mpumalanga	0.015	(0.004, 0.025)	0.01	0.002	(−0.008, 0.012)	0.68
Northern Cape	0.016	(0.002, 0.029)	0.03	0.002	(−0.011, 0.015)	0.72
Northwest	0.007	(−0.002, 0.016)	0.11	−0.004	(−0.013, 0.005)	0.40
Western Cape	−0.002	(−0.015, 0.011)	0.78	0.003	(−0.002, 0.008)	0.21

†Calculations of percent quarterly change included all smear positive TB patients registered with treatment outcome data in the ETR.

## Discussion

This analysis documents the impact of a national program to trace TB patients who interrupted treatment or missed a clinic visit in South Africa. The overall percent quarterly change for all smear positive TB patients in South Africa from Q1 2007 through the end of the TB Tracer Project in Q1 2009 showed a significant decrease in default treatment outcomes and a significant increase in successful treatment outcomes among tracer subdistricts. Changes over time were significantly different between tracer and non-tracer subdistricts for treatment outcomes of default, completed, and failed. Specifically, the decreasing trend in the proportion of patients who defaulted over time was significantly greater among tracer subdistricts than non-tracer subdistricts. The proportion of patients who completed treatment also had a declining trend over the time period for each the tracer and non-tracer subdistricts; however, the slope was significantly less among the tracer subdistricts than the non-tracer subdistricts. These findings demonstrate a significant temporal association between TB tracer teams and TB treatment outcomes.

Our findings are supported by a study conducted in Kenya at clinics operated by Médeicins Sans Frontières (MSF) which demonstrated that the implementation of an active defaulter tracing system for HIV, prevention of mother-to-child transmission, and TB patients resulted in a decrease in TB patients lost to follow up [11]. Furthermore, the MSF tracing system documented a high resumption of appointments by patients and was able to establish a treatment outcome for almost 85% of patients who missed an appointment [11].

In our study, we found that the impact of the TB Tracer Project varied by province. The inconsistency in the results observed between the provinces could be attributable to a variety of factors not assessed in this analysis: differential patient and tracer subdistrict sample sizes between provinces, variability in reporting and recording of TB treatment outcomes, as well as differences in TB burden, HIV prevalence, infrastructure, socioeconomic structure and geography. Previous research has cited the relationship between the health provider and patient and the pattern of health care delivery to be significantly associated with patient default [3,7,13-18]. The differences in results between provinces may also be due to geographic migration patterns; a study of multidrug resistant TB in South Africa found that being born outside of South Africa and changing residence during treatment were both significantly associated with default from treatment [15]. Additionally, variations in staffing and in the number of tracer teams operating per health facility and per subdistrict may have affected the efficacy of the TB Tracer Project. While this analysis did not assess these qualitative issues, a parallel study is underway to determine whether the differences in impact of the TB tracer teams may be attributable to some of these factors.

The present study was unique as few other treatment default and adherence studies have been able to assess the issue both nationally and within specific country regions. However, this study is not without limitations. This was an ecological study using a non-randomized selection of tracer and non-tracer subdistricts where in inclusion in the project was based upon one of the outcomes of interest, thereby allowing for differences in case load and for possible bias in our results. The evaluation of the TB Tracer Project was requested and conducted after the completion of the project design and implementation. Many data elements necessary for an epidemiologic evaluation of the impact of this intervention were not available for analysis, including patient level information, details of tracer teams’ duties and actions, and tracer team coverage of subdistricts and/or health facilities. However, by using national programmatic data from the ETR we were able to account for baseline trajectories in modeling with national standardized surveillance data. The subdistrict was utilized as the unit of analysis for this study because it was not possible to reliably account for and categorize the tracer status for all individual health facilities. However, the level of misclassification is likely similar in both groups and therefore would not introduce a systematic bias in the data aggregated at the subdistrict level. This non-directional misclassification would have biased toward a null result of finding no difference in the outcome between tracer and non-tracer sites. Nonetheless, the differences in the proportions of TB treatment outcomes between tracer and non-tracer subdistricts both prior to and during the TB Tracer Project were inherent in the study design [12]. However, by modeling the proportion of TB treatment outcomes rather than patient counts with a large national sample, we aimed to minimize the effect of this selection bias.

This analysis was restricted to smear positive TB patients registered in the ETR with a treatment outcome recorded and therefore the results may not be representative of all TB patients who defaulted from treatment. However, we were able to capture the majority of patients in the ETR cohorts from Q1 2007 to Q1 2009. The aggregate ETR data available for this analysis limited our ability to produce a quantifiable point estimate to evaluate the effect of the tracer teams on TB treatment outcomes. Yet the data allowed us to examine the impact of the tracer teams over more than a two year period for the entire country of South Africa. Furthermore, the ability to perform a province stratified analysis to assess the effect of the intervention within each South African province allows for a deeper understanding of the underlying processes at work within the NTP in South Africa and allows for greater programmatic improvements.

The programmatic implications of patient tracing extend beyond the focus of this study. The improvements achieved in patient default observed during the TB Tracer Project were statistically significant; however, the current study did not observe a significant difference between tracer and non-tracer subdistricts for overall treatment success. It is likely that other programmatic interventions (i.e., DOTS, effective medication, adequate healthcare staffing, etc.) are necessary to extend beyond decreasing treatment default and to achieve an increase in treatment success. A multi-pronged approach is essential to reach global TB treatment targets, one component of which may be tracing patients to improve adherence in addition to other TB control strategies. While this study focused on default in smear positive TB patients, we did not have information regarding the HIV status of the patients counted in the ETR nor did we have data for smear negative TB patients. Research has found that patients undergoing HIV and TB treatment are more likely to interrupt treatment and the implications of TB treatment default for an HIV positive patient are of particular concern in a high-burden HIV setting [3,15]. We chose not to focus on MDR TB patients in this study; however, the repercussions of treatment default for MDR TB patients must be considered when evaluating the importance of a TB tracing program [15].

## Conclusion

In conclusion, this study provides important data on the efficacy of using patient tracers to improve TB outcomes in South Africa. Our results demonstrate that community mobilization of teams designated to trace TB patients may be an effective strategy to mitigate TB default rates and improve TB treatment outcomes. A parallel study by Bristow et al. is underway to assess knowledge, attitudes, challenges, and best practices regarding TB tracing activities and to elucidate discrepancies across provinces in South Africa. These results will shape future research to implement a full scale TB tracing program with ongoing monitoring and evaluation. With the synergy of the TB, MDR TB, and HIV epidemics in South Africa, the need to increase treatment success and to decrease default is paramount.

## Competing interest

The authors have no competing interests to report. The findings and conclusions in this report are those of the authors and do not necessarily represent the official position of the Centers for Disease Control and Prevention.

## Author’s contribution

LEB, LJP, AP, PS, LN, MVW, and LDM contributed to the study design. LEB and LJP designed the overall statistical analysis plan, analyzed the data, and take responsibility for the accuracy of the data analysis. LEB drafted the manuscript with assistance and input by LJP. LEB, LJP, AP, PS, LN, MVW, and LDM reviewed the findings for the interpretation of the data and the manuscript for intellectual content as well as critical review and editing. All authors read and approved the final manuscript.

## Pre-publication history

The pre-publication history for this paper can be accessed here:

http://www.biomedcentral.com/1471-2458/12/621/prepub
